# Fuel cell performance improvement via the steric effect of a hydrocarbon-based binder for cathode in proton exchange membrane fuel cells

**DOI:** 10.1038/s41598-022-18464-6

**Published:** 2022-08-17

**Authors:** Jung-Eun Cha, Won Jae Cho, Jeemin Hwang, Dong-Jun Seo, Young-Woo Choi, Won Bae Kim

**Affiliations:** 1 Hydrogen Energy Research Division, Fuel Cell Research & Demonstration Center, Korea Institute of Energy Research (KIER), 20-41 Sinjaesaengeneogi-ro, Haseo-myeon, Buan-gun, Jeollabuk-do 56332 Republic of Korea; 2 Department of Chemical Engineering, Pohang University of Science and Technology (POSTECH), 77 Cheongam-ro, Nam-gu, Pohang, Gyeongbuk 37673 Republic of Korea; 3 Clean&Science Co., Ltd., Factory 67, 3Sandan 3gil, Buk-myeon, , Jeongeup-si, Jeollabuk-do 56137 Republic of Korea

**Keywords:** Energy science and technology, Fuel cells

## Abstract

In this study, a sulfonated poly(ether sulfone) having cardo-type fluorenyl groups (FL-SPES) was investigated as a cathodic binder to improve fuel cell performance via increased the oxygen diffusion in the cathode. The maximum power density achieved by using the membrane electrode assembly (MEA) prepared with FL-SPES with a low ion exchange capacity (IEC) of 1.31 meq g^–1^ was 520 mW cm^–2^, which is more than twice as high as that of BP-SPES (210 mW cm^–2^) having typical biphenyl groups with a similar IEC. At high IEC of 1.55 meq g^–1^, the power density obtained by using BP-SPES was improved to 454 mW cm^–2^ but remained lower than that of FL-SPES. In addition, although the IEC, swelling degree, and specific resistance were similar to each other, the gas permeability of FL-SPES was improved by approximately three times compared to that of BP-SPES. The steric structure of cardo-type FL-SPES increased the free volume between the polymer backbones, leading to an increase in gas transfer. Consequently, oxygen diffusion was promoted at the cathode, resulting in improved fuel cell performance.

## Introduction

Proton exchange membrane fuel cells (PEMFCs) have attracted attention as next-generation transportation, distributed power, and portable energy sources because of their high energy density, fast start-up and response characteristics, and excellent durability. Nafion®, a representative perfluorosulfonic acid (PFSA), exhibits excellent chemical and mechanical stability and high ion conductivity and is widely used as a catalyst-electrode binder as well as a membrane for PEMFCs^1–3^. However, PFSAs exhibit significant drawbacks, such as insufficient thermomechanical stability, high cost, environmental hazards, and restricted operation temperature windows. Therefore, many studies have been conducted on the development of hydrocarbon-based materials as alternatives to PFSAs^4^.

Among the many promising hydrocarbon polymers^5–11^, sulfonated poly(ether sulfone) (SPES) is low-cost due to its simple synthesis method, has a low gas permeability due to its dense and rigid structure, and has excellent thermal stability compared to Nafion^6–8^. Although significant progress has been made in the study of using hydrocarbon polymer electrolytes as membranes^9,10^, little research has been conducted on the role of binders in catalyst layers^11–14^ because hydrocarbon polymers exhibit lower oxygen permeability coefficients than PFSA^13–15^. Because the oxygen reduction reaction (ORR) occurring at the cathode is slower than the hydrogen oxidation reaction (HOR) at the anode, the ORR at the cathode is a major cause of polarization loss and is the key factor controlling the electrochemical reaction rate in PEMFC operation^14–16^. Therefore, several studies have been conducted to improve the ORR rate using an SPES binder in the cathode catalyst layer, such as improving oxygen diffusion by controlling the catalyst microstructure, optimizing binder loading, adding phosphoric acid, and using multi-block copolymers as functional groups^12,13,17,18^.

In this study, we focused on increasing oxygen diffusion at the cathode by inducing steric structures such as cardo-type fluorenyl groups (FL-SPES)^19–21^ instead of the flat structures of biphenyl groups (BP-SPES) to increase the free volume in the chemical structure of SPES. In addition, we determined how oxygen diffusion affects fuel cell performance by conducting a single-cell test and comparing an MEA with BP-SPES and FL-SPES as a cathodic binder.

## Experimental procedure

### Preparation of the binders and the membranes

BP-SPES (with biphenyl groups) and FL-SPES (with fluorenyl groups) were synthesized by polycondensation with 4,4’-dihydroxylbiphenyl (DHBP) and 9.9-bis(4-hydroxyphenyl fluorene)(HPFL), respectively, as reported previously^4^. To decouple the effect of the structural difference and intrinsic properties such as the IEC of the ionomers, BP-SPES and FL-SPES were synthesized with similar IECs and grouped. For instance, ionomers with low IECs of 1.25 ± 0.05 meq g^–1^ were grouped and denoted BP1 and FL1, whereas those with high IECs of 1.5 ± 0.05 meq g^–1^ were named BP2 and FL2. The anodic binder was prepared by mixing, Pt/C (40 wt%, Vulcan XC-72, USA), 5 wt% Nafion ionomer solution, and IPA were mixed with an appropriate amount of deionized water in a vial. The cathodic binder solutions were prepared using a Nafion ionomer as the reference and the BP-SPES and FL-SPES polymers such as BP1, BP2, FL1, and FL2 after they were protonated. In addition, the hydrocarbon membrane (HCM) was prepared using a commercial sulfonated poly(ether sulfone)(SPES) purchased from Yanjin. This sulfonated polymer contains biphenyl groups and its degree of sulfonation is 50%. The membrane was prepared with a thickness of approximately 47 µm, which is similar to that of the commercial Nafion212 membrane (approximately 50 µm). Additional details are provided in the Supplementary information (SI).

### Characterization of BP-SPES and FL-SPES

^1^H NMR spectra were obtained using a 400 MHz Fourier Transform Nuclear Magnetic Resonance Spectrometer (FT/NMR) (JNM-EX400, JEOL, Japan) with BP-SPES and FL-SPES dissolved in deuterated dimethyl sulfoxide (DMSO-d6). Fourier Transform-Infrared Spectroscopy (FT-IR) spectra for both SPESs were examined using an FT-IR spectrometer (4100E, JASCO Deutschland GmbH, Pfungstadt, Germany) in the wavenumber range of 4000–500 cm^–1^ and a transmittance mode. Gas permeability tests were performed using a Gurley 4340 N densometer^22^. An orifice area of 6.452 cm^2^ and gas flow rate of 100 ml s^–1^ were applied under a standard pressure (1.23 kPa). For IEC measurements, H^+^-form samples soaked in 50 mL of a 3 N aqueous NaCl solution for 24 h were titrated with 0.01 M NaOH using an electronic titrator (Metrohm 848 Titrino Plus, Metrohm, Switzerland). The specific resistance of the samples (at least 1 cm × 4 cm) soaked in deionized water at 25 °C was evaluated using an electrochemical impedance spectrometer (ZIVE SP1, Korea). For the swelling ratio, the samples were sufficiently wetted in deionized water at 25 °C, and their areas and volume selling degrees were calculated. The details of the characterization are provided in the SI.

### MEA fabrication and PEMFC performance test

The MEAs were fabricated by spraying binder solutions to obtain an active area of 25 cm^2^ evenly on both sides of the Nafion 212 (Dupont, USA) membrane and hydrocarbon membrane (HCM) with biphenyl groups with a 50% degree of sulfonation. Subsequently, the platinum loading on each side of the membranes was maintained at 0.4 mg cm^–2^ (see the *MEA fabrication and fuel cell performance test* session of the SI for additional description). The fuel cell performance of the prepared MEAs was measured with hydrogen as a fuel (0.4 L min^–1^) and air as an oxidant (1.5 L min^–1^) at 80 °C and 100% relative humidity (RH). The power density curves in voltage sweeping mode and electrochemical impedance characteristics at 40 and 800 mA cm^–2^ were recorded using a potentiostat with electrochemical impedance spectroscopy (EIS) (BioLogic Science Instruments, HCP-803, France).

## Results and discussion

**Figure 1:**
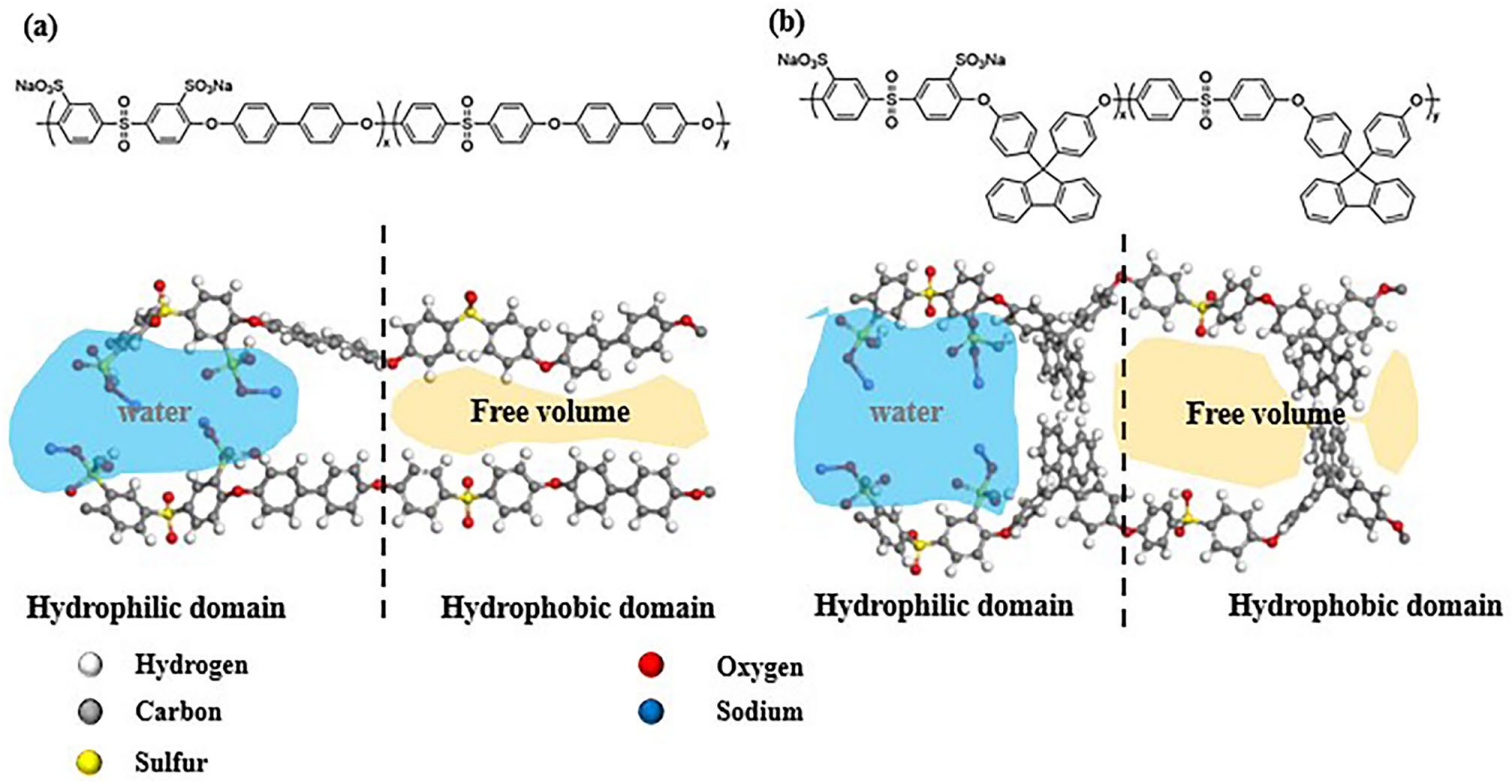
Chemical structures of the (**a**) BP-SPES composed of a flat linear structure and (**b**) FL-SPES composed of a steric structure.

Figure 1 shows the chemical structures of the BP-SPES and FL-SPES. Both SPESs were divided into hydrophilic and hydrophobic domains. Compared to BP-SPES composed of a flat linear structure (Fig. 1a), the quaternary carbon and bulky ring structure of the cardo polymer cause structural rigidity in FL-SPES by significantly restraining the rotational motion of the polymer bonds leading to relative steric hindrance^21,23^. The rigidity enables FL-SPES to have a large free volume in the hydrophobic domain, which is advantageous for gas permeation, whereas the steric structure limits the degree of swelling in the hydrophilic domain (Fig. 1b)^19–21,23^.

Hydrocarbon-based electrolyte polymers typically require significantly higher amounts of sulfonic acid functional groups (-SO_3_H) per repeating unit to achieve ion conductivity similar to that of Nafion, resulting a higher IEC. The obscure separation between the hydrophobic and hydrophilic phases in BP-SPES due to the densely located functional groups and flexible backbone produces narrow and unconnected domains that hinder proton conductivity and yield a higher swelling degree^15,24^. In contrast, the attractive force between the cardo-type fluorenyl groups in the two different FL-SPES–bones is advantage as a cathodic binder as one compartment maintains distinct phase separation regardless of the IEC. This phase separation could be a significant factor in promoting the ORR at the cathode using the FL-SPES binder.

**Figure 2:**
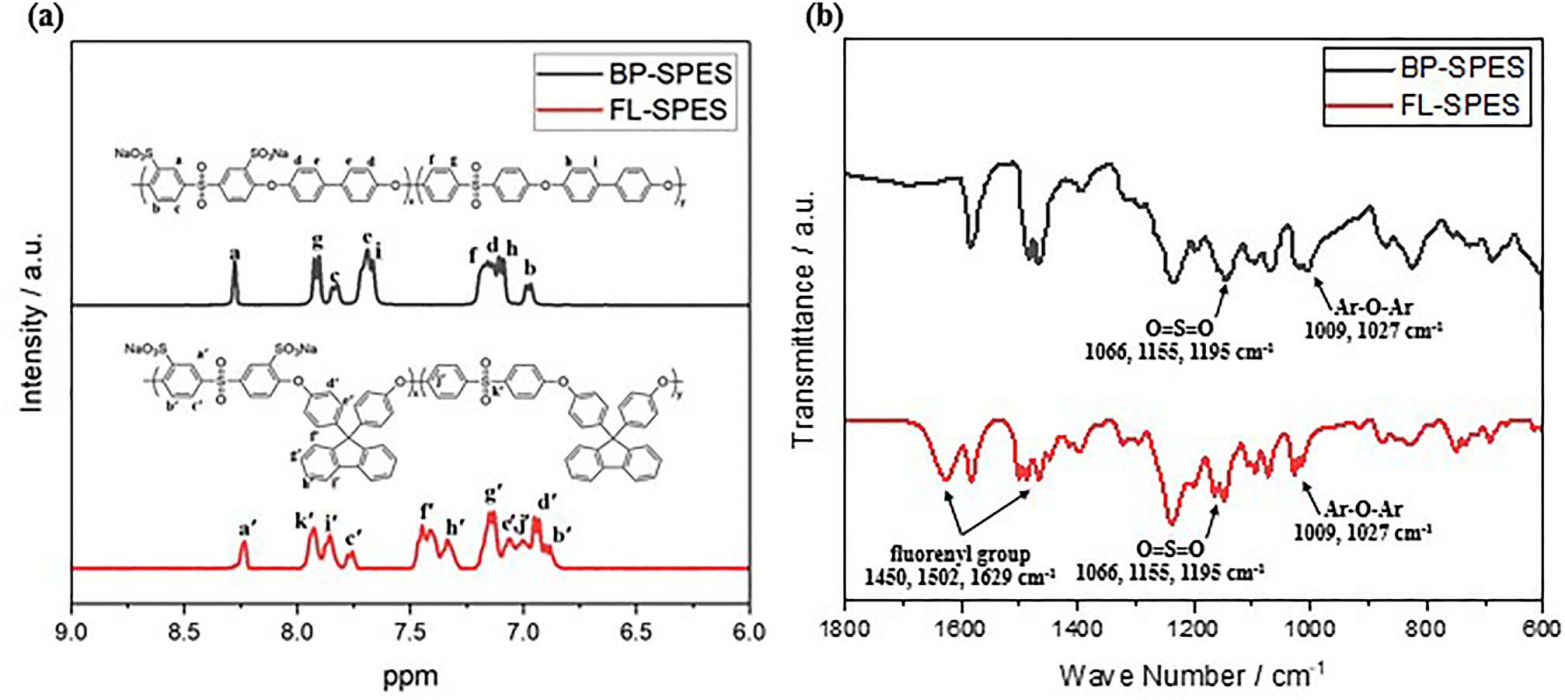
^1^H-NMR and FT-IR spectra of BP-SPES and FL-SPES.

The chemical structures of BP-SPES and FL-SPES were verified using ^1^H NMR spectroscopy. Figure 2a shows the ^1^H NMR stacked spectra of the two types of SPES with signal assignments. Marked changes in the ^1^H NMR spectrum of FL-SPES are observed compared with that of BP-SPES. New signals appear at 7.2, 7.4 and 7.83 ppm, corresponding to the protons in the fluorenyl group^10,23^.

FT-IR spectroscopy was performed to analyze the characteristic bands corresponding to the sulfonate groups in the two SPES structures (Fig. 2b). They have also been typically observed with sulfonated poly(phenylene sulfide)s synthesized using biphenyl^25^ and fluorenyl groups^10^. The stretching bands at 1009 and 1027 cm^–1^ can be assigned to Ar–O–Ar vibrations^25^. The characteristic peak for the aromatic sulfone group appear at 1155 cm^–1^, and the stretching bands at 1066 and 1195 cm^–1^ are observed due to the O=S=O vibrations of the sulfonic acid groups^26^. The stretching bands observed at 1450, 1502, and 1629 cm^–1^ can be assigned to the cardo-type aromatic rings in the fluorenyl groups of FL-SPES^23^. The ^1^H NMR and FTIR analysis results confirm that the polymerization of BP-SPES and FL-SPES employed in this study was successful.

Table 1 summarizes the key properties of the BP-SPES and FL-SPES. Low and high IECs for both BP-SPES and FP-SPES were selected for comparison (see the “Experimental procedure” section for details). The SPESs with low IECs, BP1 (1.22 meq g^–1^) and FL1 (1.31 meq g^–1^), present similar specific resistances of 40.0 and 43.4 Ω cm, respectively. As the specific resistance is the reciprocal of the proton conductivity, a high IEC can reduce the specific resistance, which can be confirmed by comparing BP2 and FL2 to BP1 and FL1. The specific resistances of BP2 and FL2 decrease to 18.2 and 28.6 Ω cm, whereas their IECs increase to 1.55 and 1.45 meq g^–1^, respectively. Both BP2 and FL2 are suitable as ion exchange membranes in PEMFCs due to their high IECs and low specific resistances; however, for cathodic binders, the swelling degree should also be considered. The degrees of water swelling in BP1 and FL1 with low IECs are 19.9% and 18.64%, respectively, similar to the values of 15–20% for Nafion ionomer (N). However, in the samples with high IECs, the swelling degree of FL2 increases only slightly, to 21.52%, whereas that of BP2 is 34.62%. These results are in good agreement with the hypothesis for the steric structure of the FL-SPES mentioned earlier.

**Table 1:** Properties of the ionomers and membranes used in this study.

Sample	Ion exchange capacity (meq g^–1^)	Specific resistance (Ω cm)	Swelling degree (%)
BP1	1.22	40.0	19.90
BP2	1.55	18.2	34.62
FL1	1.31	43.4	18.64
FL2	1.45	28.6	21.52
N ^28^	0.90	11.7	15–20
HCM	2.13	11.9	55.00
Nafion212	0.90	11.7	15.67

BP1, BP2, FL1, and FL2 indicate SPES ionomers synthesized with biphenyl and fluorenyl groups, respectively. N represents the Nafion ionomer of EW1100. HCM is a typical SPES hydrocarbon membrane with a 50% degree of sulfonation, synthesized by biphenyl groups.

As a cathodic binder should facilitate a triple phase boundary, a high swelling degree should be avoided because it can sometimes hinder oxygen diffusion under normal PEMFC operation conditions^27^. Therefore, an ideal cathodic binder requires both low specific resistance and a low swelling degree, which is paradoxical as these factors are inversely proportional. So far, Nafion is the only ionomer that exhibits these peculiar properties. In this study, FL2 showed the potential to become another ideal cathodic binder candidate.

**Figure 3:**
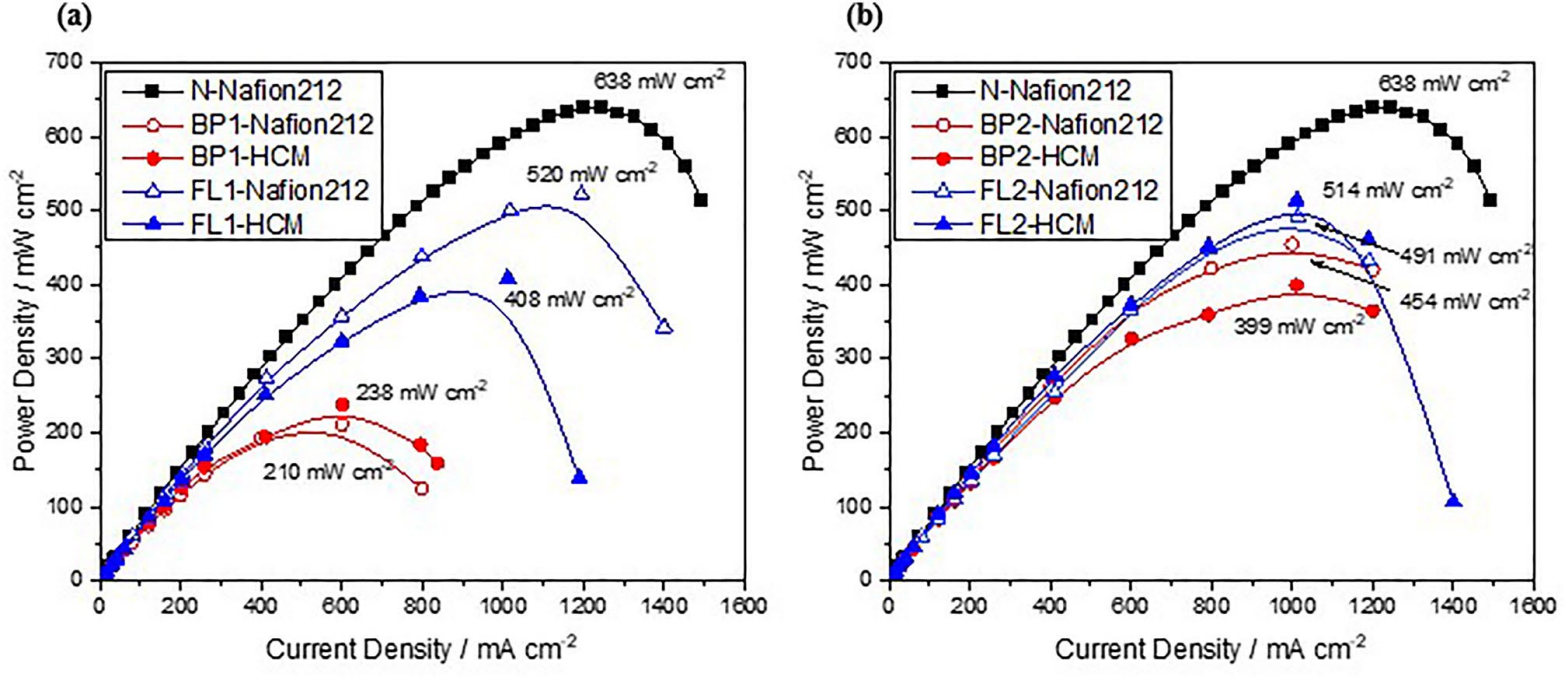
Power densities of the MEAs prepared with (**a**) BP1 and FL1 and (**b**) BP2 and FL2 as cathodic binders compared to the Nafion ionomer at a cell temperature of 80 °C and RH of 100%. N represents the Nafion ionomer of EW1100. BP1 and BP2 indicate the SPES synthesized by biphenyl groups with IECs of 1.22 and 1.56 meq g^–1^, respectively. Similarly, FL1 and FL2 indicate the SPES synthesized by fluorenyl groups with IECs of 1.31 and 1.45 meq g^–1^, respectively. The ion exchange membrane were Nafion 212 and HCM. HCM indicates a typical SPES hydrocarbon membrane with a 50% degree of sulfonation synthesized by biphenyl groups. N-Nafion212 denotes a Nafion ionomer as a cathodic binder combined with a Nafion212 membrane.

**Figure 4:**
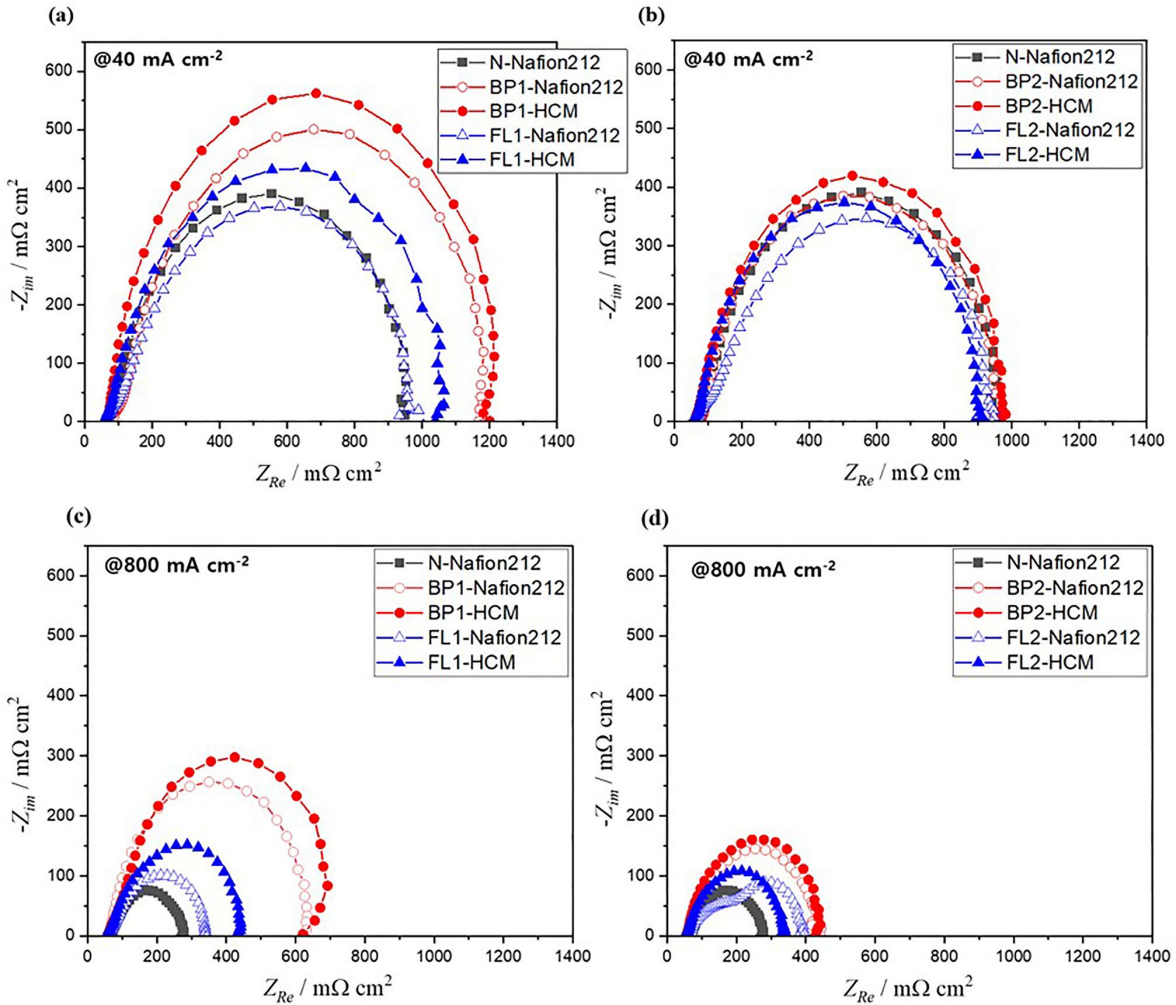
Impedance plots for the MEAs prepared with BP-SPES and FL-SPES (**a**, **b**) @ 40 mA cm^–2^ and (**c**, **d**) 800 mA cm^–2^ at a cell temperature of 80 °C with 100% RH.

In order to verify the effect of the SPES structure in depth, the gas permeability was measured. BP1 and FL1 were selected among the SPES as both have swelling degrees comparable to that of Nafion. The gas permeability measurements were performed by Gurley Precision Instruments using air to verify the free volume. The Gurley time of BP1 was three times lower (24,791 s) on average than that of FL1 (8820 s). Conversion of these values into gas permeabilities^4^ yielded 0.0051 and 0.0153 µm Pa^–1^ s^–1^ for BP1 and FL1, respectively. We assumed that the steric structure of FL-SPES with cardo-type fluorenyl groups increased the free volume between the polymer backbones to increase gas transfer. Therefore, when FL-SPES was used as the cathodic binder, oxygen diffusion was promoted at the cathode. However, it is smaller than Nafion, which has a gas permeability of 0.0241–0.0362 µm Pa^–1^ s^–1^ ^28^. This is further verified by the fuel cell performance shown in Figs. 3 and 4. Here, an HCM and Nafion212 with a similar specific resistance were used to compare the miscibility between the membrane and cathodic binder for PEMFC single-cell tests.

To obtain the fuel cell performance measurements in Fig. 3, BP-SPES and FL-SPES binders with Nafion212 and HCM as membranes were fabricated as MEAs. N was adopted as a reference at 80 °C with 100% RH. Figure 3a illustrates the maximum power densities between the groups with low IEC values. The MEA with N-Nafion212 shows the highest power density of 638 mW cm^–2^. The maximum power density values decrease in the order of FL1-Nafion212, FL1-HCM, BP1-Nafion212, and BP1-HCM as 520, 408, 238, and 210 mW cm^–2^, respectively. Notably, FL1-Nafion212 exhibits more than twice the maximum power density of BP1-Nafion212. Even when HCM is used as a membrane, the maximum power density for FL1-HCM increases by 1.7 times compared to that of BP1-HCM. As predicted above, these results confirm that the fuel cell performance is improved by the increase in free volume according to the cardo-type steric structure.

Regardless of the membrane used, MEA with BP1 binder exhibits the lowest performance. Because of the slow ORR kinetics that dominates the overall reaction, the resistance of the membrane has little effect on the fuel cell performance. In contrast, FL1 shows a clear difference in the highest power density depending on the applied membrane, which is due to the membrane resistance and slow proton conduction at the interface between the membrane and the cathodic binder. Thus, the fuel cell performance is enhanced solely by the improvement in the oxygen diffusion due to the steric structure of the binder. In addition, combination with a high-performance membrane such as Nafion212 is essential for a binder with a low IEC.

Figure 3b presents the power density of each MEA manufactured using BP2 and FL2, which have high IECs, and displays clearly different trends from those obtained with low IECs, as illustrated in Fig. 3a. Most notably, the power densities of BP2-Nafion212 and BP2-HCM are significantly increased compared with those of the corresponding BP1 binders. Even the power density of BP2-HCM (399 mW cm^–2^), which is the lowest among the high-IEC binders, is 1.67 times higher than that of BP1-HCM. The power density of BP2-Nafion212 is also increased to 454 mW cm^–2^. Besides BP1, MEA with BP2 shows a difference in power density according to the applied membrane. Nevertheless, FL2 still achieves the highest performance regardless of the membrane.

Here, the fuel cell performance of MEA with BP2 is significantly higher than that with BP1 because the increase in IEC strongly promotes ion transport in the cathodic binder to the extent rather than the impact of the oxygen diffusion resistance. Therefore, the fuel cell performances of the binders with low IECs are determined by the specific resistances^29^. Compared to FL1-Nafion212, which has the highest power density in Fig. 3a, FL2-Nafion212 yields similar values despite the increased IEC. Interestingly, even when the HCM is used, there is little degradation in fuel cell performance due to the membrane and interfacial resistance. Hence, the improved ORR rate and acceleration of proton movement are attributable to the structural improvement of FL-SPES and enhanced proton conduction (i.e., decreased specific resistance) due to the high IEC. This is also the reason that the fuel cell performance differs depending on the membrane in MEA with BP2, which has a high IEC, unlike BP1.

To explain the fuel cell performance of each MEA further, in situ EIS was conducted at 40 and 800 mA cm^–2^ under the fuel cell operating conditions to verify the cause of voltage loss from the perspectives of kinetic resistance (Fig. 4a and b) and mass transfer resistance (Fig. 4c and d), respectively. The high-frequency intercept (the first intercept) at Z_Re_ represents the ohmic resistance, which includes the interface contact and membrane resistances^30,31^. The MEAs formed by the BP and FL binders show comparable ohmic resistances around 60–70 mΩ cm^2^ in Fig. 4a–d. The overall cathode resistance including the membrane, interface, and charge and mass transfer resistances corresponds to the low-frequency intercept (the second intercept) of the Nyquist plot^32^, where N-Nafion212 demonstrates the lowest resistance (938 mΩ cm^2^ @40 mA cm^–2^ and 281 mΩ cm^2^ @800 mA cm^–2^), which increases in the order of FL and BP regardless of the membrane. In addition, the semicircle diameter between the high- and low-frequency intercepts represents the charge-transfer resistance related to the electrochemical reaction^30^. In Fig. 4, BP1-Nafion212 and BP1-HCM show the largest transfer resistance, indicating that the electrochemical reaction due to the oxygen transfer resistance in the fuel cell is slowed, and thus the power density is reduced (see the *EIS analysis* session of the **SI** for more description). Meanwhile, the charge and mass transfer resistance of the MEA prepared with the FL1 binder is significantly lower than that when BP1 is employed as shown in Fig. 4c. The resistance of the MEA with BP2 is significantly reduced compared to that of the MEA with BP1, accelerating ion transport in the cathodic binder with a high IEC and resulting in increased power density (Fig. 4d). These results further emphasize that the cathodic binder for improving the electrochemical activity at low IEC should have a structure with a high free volume and that when the IEC of the binder increases, the steric structure effect decreases.

## Conclusion

This paper proposed a new cathodic binder approach that can overcome the limitation of oxygen diffusion for hydrocarbon ionomers by using a cardo-type steric structure and can serve as an alternative to PFSA. To verify this approach, the IECs, specific resistances, swelling degrees, power densities, and EISs of FL-SPES and BP-SPES were measured and compared with each other. Interestingly, the ratio describing the increase in swelling degree due to the IEC increase for FL-SPES was significantly lower than that for BP-SPES. In addition, the gas permeability of FL-SPES was improved by approximately three times compared to that in the case of BP with a similar swelling degree. These results agree well with the hypothesis that the steric structure of FL provides a free volume in the hydrophobic domain while limiting the swelling degree in the hydrophilic domain. Considering the low-IEC samples, the maximum power density of FL1 was at least 1.7 times higher than that of BP1. In particular, that of FL1-Nafion212 was more than twice as high as that of BP1-Nafion212. This finding shows that the fuel cell performance was enhanced solely by the improvement in oxygen diffusion due to the steric structure of binder and that combination with a high-performance membrane is essential for a binder with a low IEC. Among the high-IEC samples, the MEA with FL2 as a binder exhibited the highest performance regardless of the membrane used even though the power density of BP2 was significantly increased compared to that of the BP1 binders. This result can be attributed to the improved ORR rate caused by the decrease in the oxygen transfer resistance in the cathode and the facilitated proton movement at the interface between the membrane and cathode due to the combined synergy of the high IEC and the structural benefit of FL-SPES. Therefore, the cardo-type fluorenyl group presented in this report proved to have sufficient potential as a cathode binder for fuel cell performance improvement. Studies of the application and characteristics of steric structure binders under various fuel cell operating conditions should be conducted in the future.

## Supplementary Information

Supplementary Information.

## Data Availability

The datasets used and/or analysed during the current study available from the corresponding author on reasonable request.
