# Supplementary material for: Fuel cell performance improvement via the steric effect of a hydrocarbon-based binder for cathode in proton exchange membrane fuel cells
Source: Sci Rep. 2022 Aug 17;12:14001. doi: 10.1038/s41598-022-18464-6 (PMC9386007; doi:10.1038/s41598-022-18464-6)
Supplement: Supplementary file 1 — Supplementary Information. [file 41598_2022_18464_MOESM1_ESM.docx]

**Scientific Reports**

**Supplementary Information**

**Fuel cell performance improvement via the steric effect of a hydrocarbon-based binder for cathode in proton exchange membrane fuel cells**

Jung-Eun Cha, Won Jae Cho, Jeemin, Hwang, Dong-Jun Seo, Young-Woo Choi, and Won Bae Kim

**Supplementary Information**

***Materials*** High purified, commercially available, 4,4’-dihydroxybiphenyl (DHBP) and Bis(4-chlorophenyl) sulfone (DCDPS) were purchased from Sigma-Aldrich (USA). Bis(4-chlorophenyl-3-sulfophenyl) sulfone disodium salt (SDCDPS) was sourced from Yanjin (China). 9,9-bis(4-hydroxyphenyl fluorene)(HPFL) was obtained from TCI (Japan). Toluene, potassium carbonate, and N,N-dimethylacetamide (DMAc), and isopropyl alcohol (IPA) were purchased from Sigma-Aldrich. Nafion ionomer having equivalent weight 1100 for a reference binder. Nafion212 membrane were purchased from Dupont (USA) and the hydrocarbon membrane (HCM) was prepared using a commercial sulfonated poly(ether sulfone) (SPES) having biphenyl groups with a degree of sulfonation of 50% provided by Yanjin.

***Preparation of SPES having biphenyl groups (BP-SPES) and having fluorenyl groups (FL-SPES)*** BP-SPES having biphenyl groups was synthesized by polycondensation with DHBP as reported in a previous paper^[1]^. At this time, the IEC was changed by adjusting the degree of sulfonation according to the change in the molar ratio of DCDPS and SDCDPS. FL-SPES having fluorenyl groups instead of biphenyl groups was polymerized with HPFL through a similar procedure. The molar ratio between DCDPS and SDCDPS was adjusted to obtain different IECs. Therefore, BP-SPES and FL-SPES with similar IECs were polymerized to compare the differences in their properties and fuel cell performances. For instance, whereas the molar ratio of DCDPS:SDCDPS was 0.7:0.3 based on 1 mol of DHBP, BP1-SPES (simply BP1) with an IEC of 1.22 meq. g^–1^ was polymerized. Meanwhile, when the molar ratio of DCDPS:SDCDPS based on 1 mol of HPFL was 0.6:0.4, it was possible to synthesize FL1-SPES (simply FL1) withf an IEC of 1.31 meq. g^–1^. Similarly, BP2, with an IEC of 1.55 meq. g^–1^ was obtained by changing the molar ratio of DCDPS:SDCDPS to 0.6:0.4 for 1 mol of DHBP, and the DCDPS:SDCDPS molar ratio of 0.5:0.5 for 1 mol of HPFL led to FL2 with an IEC of 1.45 meq. g^–1^.

***Preparation of electrode binder solutions*** For the anodic binder, Pt/C (40 wt%, Vulcan XC-72, USA) and deionized water were mixed in an appropriate vial. A 5 wt% Nafion ionomer solution and IPA were mixed in a beaker and poured into the vial under a nitrogen atmosphere. The resulting slurry was sonicated for 60 min and then stirred overnight. The preparation of the cathodic binder solution using a Nafion ionomer as a reference was the same as that used for the anodic binder solution. However, for BP-SPES and FL-SPES, polymers such as BP1, BP2, FL1, and FL2 were firstly protonated in 2 N HCl for 12 h and washed several times using deionized water. Next, each polymer was dissolved in DMAc at 10wt% and mixed with IPA : deionized water ratio of 1:1 to prepare cathodic binder solutions.

***Preparation of HCM*** The hydrocarbon membrane (HCM) was prepared using a commercial sulfonated poly(ether sulfone)(SPES) provided by Yanjin. The SPES contains biphenyl groups with a 50 % degree of sulfonation. One equivalent of SPES and 4 mL of DMAc (N, N-dimethylacetamide, Sigma Aldrich) were added together, and the mixed solution was stirred for 2 h at 25 °C. The HCM was prepared by casting the mixed solution on a 10 × 10 cm^2^ glass plate, followed by sequential drying steps at 80 °C for 12 h and at 120 °C for 5 h to completely evaporate the solvent. The HCM was then peeled off from the glass plate and the edges were cut and discarded. The membrane was prepared with a thickness of approximately 47 µm, which is similar to that of the commercial Nafion212 membrane (approximately 50 µm).

***Physicochemical properties*** The measurement of IEC, specific resistance, and water swelling ratio for the synthesized polymers was performed after being made into membranes. For IEC measurements, H^+^-form samples soaked in 50 mL of a 3 N aqueous NaCl solution for 24 h were titrated with 0.01 M NaOH using an electronic titrator (Metrohm 848 Titrino Plus, Metrohm, Switzerland). Its value is calculated by

$\mathrm{IEC}\left( \text{meq}\text{·}\text{g}^{-1} \right)=\frac{\text{V}_{\text{NaOH}}\text{C}_{\text{NaOH}}}{\text{M}_{\text{dry}}},$ (1)

where V_NaOH_ is the volume of the consumed NaOH solution (mL), C_NaOH_ is the molar concentration of the NaOH solution (M), and M_dry_ is the mass of the dried membrane (g). The theoretically calculated IEC values were then compared with the experimentally measured IEC values of the synthesized BP-SPES and FL-SPES (Table S1). The experimentally measured IEC values of the synthesized BP-SPES and FL-SPES differ slightly depending on the degree of sulfonation. However, they are close to more than 90 % of the theoretically calculated IEC values. The resulting values were meaningfully applied to analyze and discuss the correlations among the specific resistance, swelling degree, and fuel cell performance in this study.

Table S1. IEC of the hydrocarbon ionomers and membrane used in this study.

| Sample | Ion exchange capacity (meq. g^–1^) | |
| --- | --- | --- |
|  | Experimentally measured | Theoretically calculated |
| BP1 | 1.22 | 1.34 |
| BP2 | 1.55 | 1.72 |
| FL1 | 1.31 | 1.27 |
| FL2 | 1.45 | 1.55 |
| HCM | 2.13 | 2.08 |

Note: BP1 and BP2, FL1 and FL2 indicate SPES ionomers synthesized with biphenyl and fluorenyl groups, respectively. HCM is a typical SPES hydrocarbon membrane with a degree of sulfonation of 50% and contains biphenyl groups.

The specific resistance of Nafion212 and HCM (at least 1 cm × 4 cm) soaked in deionized water at 25 °C was evaluated using an electrochemical impedance spectrometer (ZIVE SP1, Korea). The specific resistances of BP1, BP2, FL1, and FL2, which were used as the binders, were also measured by the same method after preparing thin films with a similar thickness (approximately 50 µm).

The specific resistance can be defined as;

$\rho\left( \text{Ω}\text{·}\text{cm} \right)=\text{Λ}\frac{\text{A}}{\text{d}}$ , (2)

where $\text{Λ}$ is a measured resistance, A and d mean a specific membrane area and a membrane thickness, respectively. For the swelling ratio, the samples were sufficiently wetted in deionized water at 25 °C, and their areas and volume swelling degrees were calculated by;

$\text{swelling ratio }\left( \text{\%} \right) = \frac{\text{W}_{\text{m.v.}}\text{-}\text{D}_{\text{m.v.}}}{\text{D}_{\text{m.v.}}}\times100,$ (3)

^1^H NMR spectra were obtained using a 400MHz FT/NMR spectrometer (JNM-EX400, JEOL, Japan) with BP-SPES and FL-SPES dissolved in deuterated dimethyl sulfoxide (DMSO-d6). FT-IR spectra for both SPESs were examined using an FT-IR spectrometer (4100E, JASCO Deutschland GmbH, Pfungstadt, Germany) in the wavenumber range of 4000-600 cm^–1^ and a transmittance mode.

Gas permeability tests were performed using a Gurley 4340N densometer ^[2]^. The orifice area of 6.452 cm^2^ and gas flow rate of 100 ml s^–1^ were applied under a standard pressure (1.23kPa). The gas permeability was calculated by

$\text{Permeability} \left( \text{μm} \text{Pa}^{-1}\text{ }\text{sec}^{-1} \right)\text{=} \frac{\text{u}}{\text{A}\text{·}\text{P}\text{·}\text{t}}$ (4)

Where u is the actual gas volume, A is the test area of the orifice, P is the mean pressure difference and t is Gurley time.

***MEA fabrication and fuel cell performance test*** To verify the effect of the cathodic binder, the MEAs were fabricated by spraying binder solutions to obtain an active area of 25 cm^2^ evenly on both sides of the membranes, i.e., the Nafion 212 membrane and the HCM, respectively. Subsequently, the platinum loading on each side of the membranes was maintained at 0.4 mg cm^-2^. In the field of fuel cell research, the oxygen transfer resistance by the ionomer to the catalyst surface is a critical issue in the case of an ultra-low Pt catalyst loading. Even for a high Pt catalyst loading, the oxygen transfer resistance may not be negligible when a new ionomer is employed in the catalyst layer. The ink, which includes the catalyst, support, ionomer, and solvents, must be prepared before the typical decal-transfer or spraying of the ink for the preparation of the MEA. This typical fabrication process induces a thick layer of ionomer on the catalyst surface, which basically occurs because the ionomer (e.g. Nafion) engages in stronger bonding on Pt than on the carbon supports, thereby increasing the thickness of the ionomer on the Pt surface to induce a high oxygen transfer resistance. Therefore, the typical method is limited to improving the MEA performance in the mass transfer region because of the thickness of the ionomer. Apart from this, determining the optimal conditions under which to fabricate the ink with the commercial Nafion and Pt/C (e.g. 0.4 mg cm^–2^ Pt loading) required considerable time and effort ^[3]^. In particular, employing a new ionomer makes it necessary to find new ink fabrication conditions with the appropriate ratio of Pt loading and ionomer to satisfy a lower oxygen transfer resistance ^[4]^. Accordingly, the results of our study on the oxygen transfer resistance even at a high Pt loading can be crucial when new ionomers instead of Nafion are employed although we followed the proven fabrication method. The results of our study are highly trustable because we used a commercial catalyst containing 40 wt.% of Pt/C manufactured by Vulcan (USA) and the values of the electrochemical surface area (ECSA) are also comparable to those in the literature ^[5],[6]^, evaluated at 35.6, 27.5, and 29.5 m^2^ g^–1^ for N-Nafion212, BP2-HCM, and FL2-HCM, respectively, as shown in Fig. S1. The ECSAs for those MEAs were measured by a cyclic voltammetry in the range of 0-1.2 V. The scan rate was 10 mV s^–1^, the hydrogen flow rate at the anode was 400 ml min^-1^, and the nitrogen flow rate at the cathode was 1500 ml min^-1^.

The fuel cell performances of the prepared MEAs was measured with hydrogen as a fuel (0.4 L min^–1^) and air as an oxidant (1.5 L min^–1^) at 80 °C and with 100% relative humidity (RH). The power density curves in voltage sweeping mode and electrochemical impedance characteristics at 40 mA cm^-2^ and 800 mA cm^-2^ were recorded using a potentiostat with electrochemical impedance spectroscopy (EIS) (BioLogic Science Instruments, HCP-803, France).

Figure S1. Cyclic voltammograms of the MEAs with N-Nafion212, BP2-HCM, and FL2-HCM

***EIS analysis*** The impedance spectra could be mainly interpreted with ohmic resistance, charge and oxygen transfer resistance, and parallel capacitance. Figure S2 presents an equivalent circuit model proposed in this study. Where, R_ohm_ means the ohmic resistance raised by the membrane and its interface contact with both electrodes. Under the premise that the hydrogen transfer and charge transfer resistance at the anode are negligibly small, R_ct_ is the charge transfer resistance mainly caused by ORR. R_mt_ indicates the mass (oxygen) transfer resistance influenced by the morphology of the cathode. Q_1_ and Q_2_ are constant phase elements at R_ct_ and R_mt_, respectively.


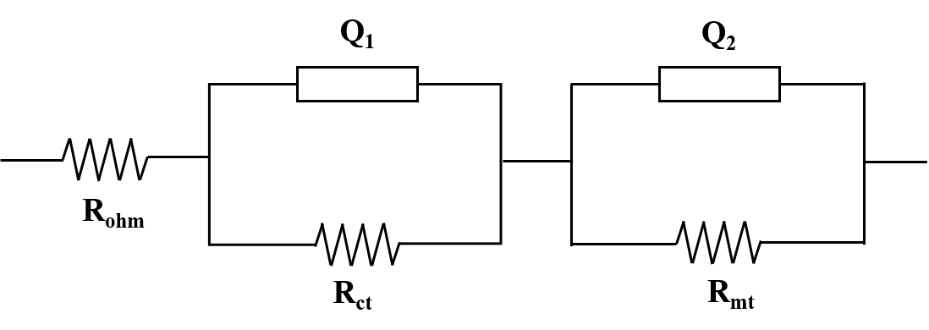


Figure S2. The equivalent circuit (EC) model proposed in this study

The charge and oxygen transfer impedance appear closely mixed due to the relative values of resistances or capacitances (Figure S3). However, the variations of Rohm, Rct, and Rmt were separated using the fitting result, close to the actual impedance measurement, derived from the EC model using the various binder as shown in Figure 4. The ohmic resistances, which are determined by the membrane, were reasonably comparable around 60–70 mΩ cm^2^ due to the similar properties of Nafion212 and HCM used. Furthermore, as shown in Figure S4, in the low current density region, the charge transfer resistance was dominant, whereas in the high current density region, the effect on the resistance was different depending on the type of binder used. However, while the charge transfer resistance for all binders in the low current density region was similar, BP1 showed the highest mass transfer resistance.

Ideally, at low current densities, the charge transfer resistance can be the same or similar regardless of the type of binder used (Figure S4(b)). However, the behaviors of the kinetic resistance can be different from the type of binder in real empirical environments, which can be estimated as a non-Fikian phenomenon. The non-Fickian diffusion is known as any form of diffusion that does not obey Fickian laws. In non-Fickian diffusion, a sharp boundary can be observed that separates the highly swollen region from the dry, amorphous region. The sharp front of the non-Fickian diffusion moves into the polymer at a constant rate so that the amount of absorbed fluid increases linearly with time^[7]^. For instance, the decrease in the kinetic activity at the Pt surface poisoned by benzene derived from the backbone of the hydrocarbon binder leads to an increase in the kinetic resistance. Many studies^[[8-10]]^ have reported that Pt poisoning by benzene rings derived from the backbone of the polymer can also reduce the kinetic activity when aromatic hydrocarbon polymer electrolytes are used as an electrode binder (particularly cathodic binder). Otherwise, when the hydrocarbon binder layers covering the Pt surface due to uncertainty of the polymer matrices have different thicknesses, the kinetic resistance can be dominated by a non-Fickian behavior depending on the type of binder because oxygen transfer resistance cannot be neglected even at the low current density (Figure S4(a)). Thus, it will need to be clarified through many studies in the future. It is obviously focused on reducing the oxygen transfer resistance with a new hydrocarbon binder in this study. In other words, we found that it is possible to reduce the oxygen diffusion resistance by the cardo-type steric structure.

*
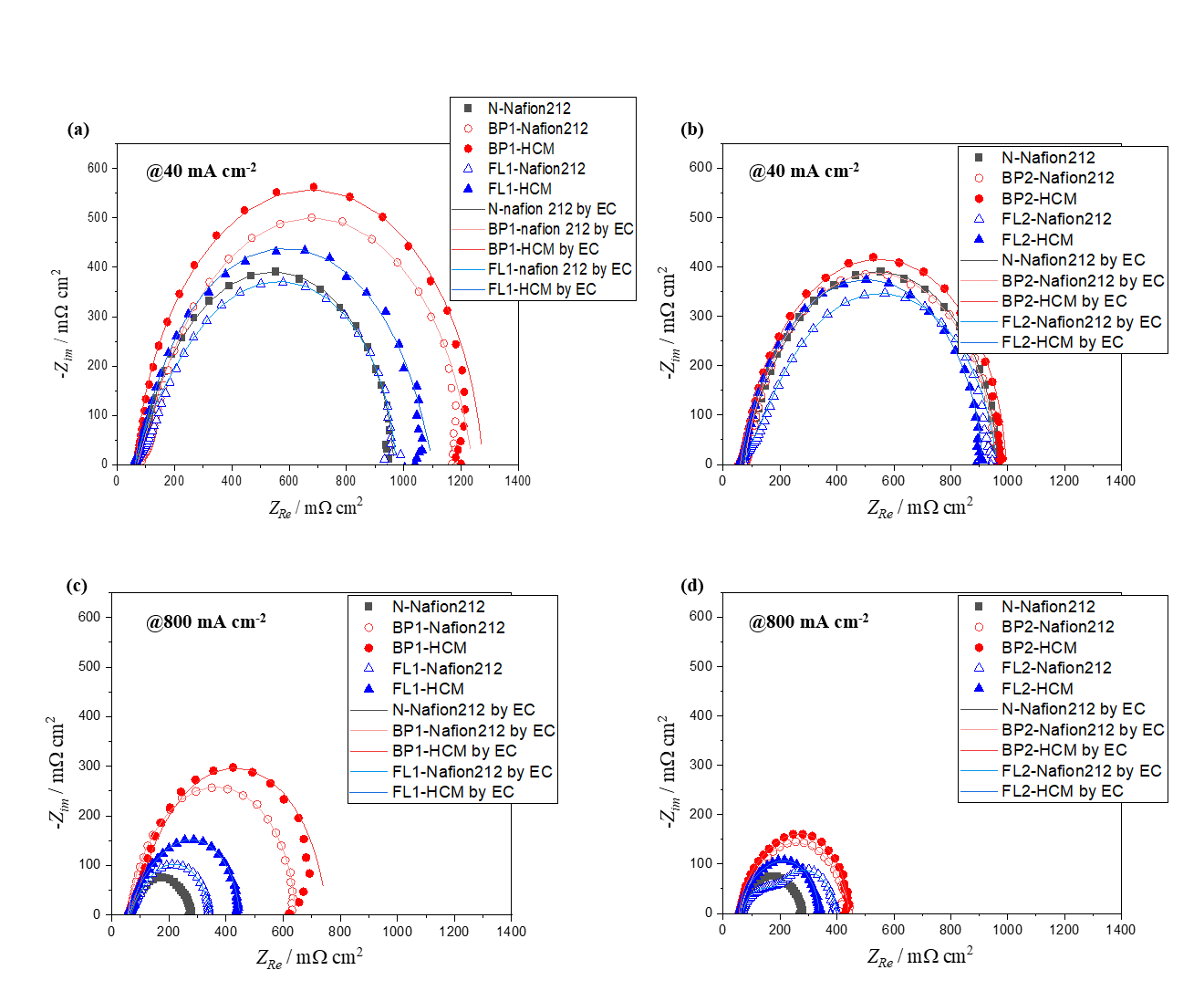
*

Figure S3. The comparison of EIS results between the experimentally measured and the calculated impedances by fitting using the EC model for the MEAs prepared with BP-SPES and FL-SPES.

*
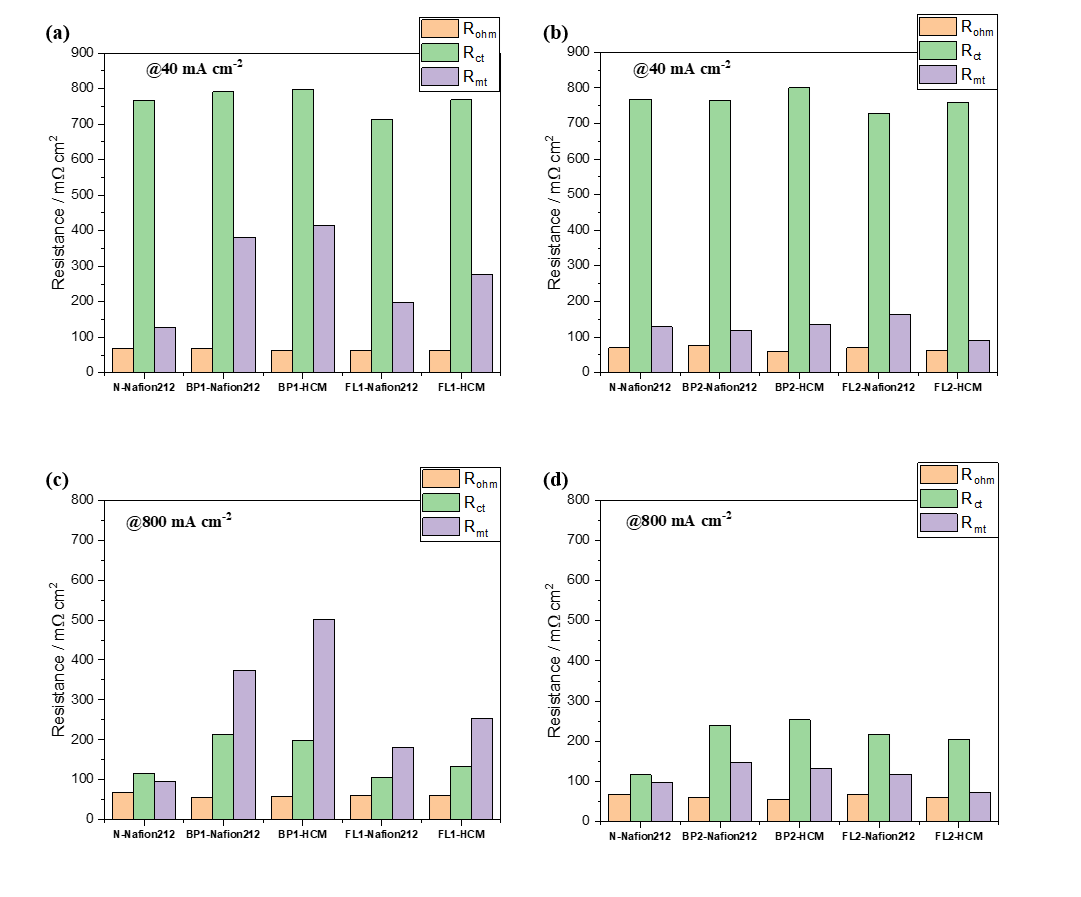
*

Figure S4. The resulting resistances derived from EIS analysis using the EC model for the MEAs prepared with BP-SPES and FL-SPES.

**References**

1 Jang, S., Yoon, Y.-G., Lee, Y.-S. & Choi, Y.-W. One-step fabrication and characterization of reinforced microcomposite membranes for polymer electrolyte membrane fuel cells. *Journal of Membrane Science* **563**, 896-902, doi:10.1016/j.memsci.2018.06.060 (2018).

2 Cai Qi, X. R., Wu Shuqiu,Chen Changbin,Mo Haibin,Lei Caihong,Li Liangbin,Zhihong Li. Influence of annealing temperature on the lamellar and connecting bridge structure of stretched polypropylene microporous membrane. *polymer international* **64**, 446-452, doi:https://doi.org/10.1002/pi.4828 (2014).

3 Jung, C.-Y. *et al.* Engineering ionomer homogeneously distributed onto the fuel cell electrode with superbly retrieved activity towards oxygen reduction reaction. *Applied Catalysis B: Environmental* **298**, 120609, doi:https://doi.org/10.1016/j.apcatb.2021.120609 (2021).

4 Nguyen, H., Klose, C., Metzler, L., Vierrath, S. & Breitwieser, M. Fully Hydrocarbon Membrane Electrode Assemblies for Proton Exchange Membrane Fuel Cells and Electrolyzers: An Engineering Perspective. *Advanced Energy Materials* **12**, doi:10.1002/aenm.202103559 (2022).

5 Pu, X., Duan, Y., Li, J., Ru, C. & Zhao, C. Understanding of hydrocarbon ionomers in catalyst layers for enhancing the performance and durability of proton exchange membrane fuel cells. *Journal of Power Sources* **493**, 229671, doi:https://doi.org/10.1016/j.jpowsour.2021. 229671 (2021).

6 Guo, J. *et al.* Protic ionic liquid-grafted polybenzimidazole as proton conducting catalyst binder for high-temperature proton exchange membrane fuel cells. *Polymer Testing* **96**, 107066, doi:https://doi.org/10.1016/j.polymertesting.2021.107066 (2021).

7 D. De Kee, Q. L., J. Hinestroza. Viscoelastic (Non-Fickian) Diffusion. *THE CANADIAN JOURNAL OF CHEMICAL ENGINEERING* **83** (2005).

8 Byungchan Bae, T. Y., Kenji Miyatake, Makoto Uchida, Hiroyuki Uchida, Masahiro Watanabe. Sulfonated Poly(arylene ether sulfone ketone) Multiblock Copolymers with Highly Sulfonated Block. Fuel Cell Performance. *J. Phys. Chem. B* **114**, 10481-10487, doi:10.1021/jp1052908 (2010).

9 Nguyen, H. *et al.* Hydrocarbon-based Pemion™ proton exchange membrane fuel cells with state-of-the-art performance. *Sustainable Energy & Fuels* **5**, 3687-3699, doi:10.1039/D1SE00556A (2021).

10 Reshetenko, T. V. & St-Pierre, J. Study of the aromatic hydrocarbons poisoning of platinum cathodes on proton exchange membrane fuel cell spatial performance using a segmented cell system. *Journal of Power Sources* **333**, 237-246, doi:10.1016/j.jpowsour.2016.09.165 (2016).
